# Response: Commentary: Manifold Routes to a Nucleus

**DOI:** 10.3389/fmicb.2019.02585

**Published:** 2019-11-08

**Authors:** Anthony M. Poole, Heather L. Hendrickson

**Affiliations:** ^1^School of Biological Sciences, University of Auckland, Auckland, New Zealand; ^2^School of Natural and Computational Sciences, Massey University, Auckland, New Zealand

**Keywords:** planctomycetes, nucleus, compartmentation, *Gemmata obscuriglobus*, eukaryogenesis

In a recent paper published in this journal (Hendrickson and Poole, 2018), we discussed and explored the evolutionary implications of emerging observations from prokaryotic systems, where forms of genetic compartmentation have been identified or proposed (Fuerst and Sagulenko, 2011; Sagulenko et al., 2014; Spang et al., 2015; Chaikeeratisak et al., 2017a,b; Zaremba-Niedzwiedzka et al., 2017). Our main aim was to consider how these observations might help shed light on the question of the origin of the eukaryote nucleus. The issue for understanding the origin of the nucleus is that it can be tricky to work on something that appears to be a singular event in evolution. This is because there is a temptation to view singular events as necessitating some type of special circumstance or one-off mechanism. Our paper aimed to address this by indicating how a range of data emerging from the study of diverse prokaryote systems might provide insight into the origins of the eukaryote nucleus, not because of a direct evolutionary relationship with eukaryotes but because they suggest that compartmentation of genetic material may not have evolved only once in the history of life. This in turn opens up the question of the origin of eukaryote nucleus to a much a broader question: what might drive the evolution of genetic compartmentation?

Jogler et al. (2019) recently published a commentary in response to our paper in which they took no issue with any of the key points of our paper. Instead, they criticized the way we discussed results concerning the fascinating bacterium, *Gemmata obscuriglobus*, and cited additional references, mostly from their own labs. *G. obscuriglobus* is relevant to our discussion of genetic compartmentation because it is the best-studied member of a phylum with members that have been reported as possessing a compartment with similarities to the eukaryote nucleus (Fuerst, 2005; Lee et al., 2009; Fuerst and Sagulenko, 2011; Sagulenko et al., 2014). In addition, other publications have reported features of *G. obscuriglobus* that are normally associated with eukaryotes, including endocytosis-like processes (Lonhienne et al., 2010), the separation of transcription and translation (Gottshall et al., 2014), and the presence of structures that resemble the nuclear pore complex (Sagulenko et al., 2017). We acknowledged that the issue of whether there is a nucleus-like compartment in *Gemmata* and related bacteria is a “matter of ongoing debate” and respectfully disagree with Jogler and colleagues' conclusion (Jogler et al., 2019) that this possibility has been disproven. For example, while one electron tomography study (Acehan et al., 2014) using plastic embedding concluded that *G. obscuriglobus* is neither compartmentalized or nucleated, a subsequent cryo-electron tomography study from an independent group (Sagulenko et al., 2014) supported compartmentalization. Similarly, Jogler and colleagues' claim (Jogler et al., 2019) that structures from the internal membrane of *Gemmata* that resemble the eukaryote nuclear pore complex (Sagulenko et al., 2017) are instead crateriform structures found on the exterior of *G. obscuriglobus* cannot account for the observations that proteins associated with these pores are exclusive to inner membranes (Sagulenko et al., 2017), and that antibodies raised against them are both specific and localized exclusively to the inner membranes. The field is still working toward a full characterization and understanding of the unusual membrane architecture (and associated biology) of *Gemmata* so it is premature to refer to a paradigm shift as having taken place.

With characterization of the cellular architecture of this fascinating bacterium a subject of ongoing research, we are open to data that could show that Jogler and colleagues' interpretation is partially or even wholly correct. However, given the main focus of our paper, there seems to be limited value in weighing in on extensive debate on this topic when it was not germane to the primary point of our article. Indeed, the key point of our paper is wholly unaffected by Jogler and colleagues' concerns. It would have been utterly remiss of us to have discussed non-eukaryotic cases of genetic compartmentation and completely ignored the extensive literature on *Gemmata*.

## Author Contributions

All authors listed have made a substantial, direct and intellectual contribution to the work, and approved it for publication.

### Conflict of Interest

The authors declare that the research was conducted in the absence of any commercial or financial relationships that could be construed as a potential conflict of interest.
